# Adolescent Exposure to Methylphenidate Increases Impulsive Choice Later in Life

**DOI:** 10.3389/fnbeh.2017.00214

**Published:** 2017-10-31

**Authors:** Zarish Abbas, Arwen Sweet, Giovanni Hernandez, Andreas Arvanitogiannis

**Affiliations:** ^1^Department of Psychology, Center for Studies in Behavioral Neurobiology, Concordia University, Montreal, QC, Canada; ^2^Groupe de Recherche sur le Système Nerveux Central, Faculté de Pharmacie, Université de Montréal, Montreal, QC, Canada; ^3^Groupe de Recherche sur le Système Nerveux Central, Département de Pharmacologie et Physiologie, Université de Montréal, Montreal, QC, Canada; ^4^Département de Neuroscience, Université de Montréal, Montreal, QC, Canada

**Keywords:** ritalin, delay discounting, impulsivity, adolescence, rats, long-term effects

## Abstract

**Background:** The psychostimulant methylphenidate (MPH) is known to temporarily reduce impulsive choice and promote self-control. What is not sufficiently understood is how repeated treatment with MPH affects impulsive choice in the long run, and whether any such effect is contingent on exposure at certain developmental stages.

**Methods**: Using an animal model for impulsive choice, we examined first whether giving MPH through early adolescence alters delay discounting, an operational measure of impulsive choice, later in adulthood. We then tested whether equivalent long-term effects are observed if exposure to the drug occurred during adulthood. Starting on postnatal day 25 or postnatal day 60, male rats received one of a range of doses of MPH for 10 consecutive days. Twenty-six days later, all rats were trained to choose between a lever that produced a small immediate reward and a lever that produced a large reward after a range of delays.

**Results**: Rats showed a long-term decrease in the selection of the delayed larger reward when treated with moderate doses of MPH during early adolescence, but not when treated with the lower or higher doses. In contrast, no differences were observed in the selection of the delayed larger reward in animals that were treated with various doses of MPH during adulthood.

**Conclusions**: Our findings suggest effects of MPH on impulsive choice that are contingent on dosage and on the developmental period of exposure. When administered during adolescence, moderate doses of MPH increase impulsive choice long after the end of treatment, whereas these same doses administered during adulthood were without effect

## Introduction

Time is money. A payout depreciates in value the more time it takes to earn it, to the point where a smaller, but more timely payout starts to look more worthwhile. What is true for money is also true for other rewards, be it a glass of wine, a cigarette, or a sugary snack: if a larger reward takes too long to earn, a smaller, quicker one wins out. For some individuals, this critical point is reached more quickly; they are said to show *impulsivity*. Others will wait longer for the same reward, showing *self-control* (Rachlin et al., 1972; Ainslie, 1975).

While these traits tend to be stable, psychostimulant drugs can dislodge and shift them. One such drug, methylphenidate (MPH), temporarily slows the erosion of reward value in humans (Pietras et al., 2003; Shiels et al., 2009) and other animals (Pitts and McKinney, 2005; van Gaalen et al., 2006), effectively curbing impulsivity and imposing self-control. As such, it is prescribed under trade names like Ritalin^©^ to treat impulse-control disorders, and is commonly used illicitly by adolescents to improve their academic performance (Low and Gendaszek, 2010; Zosel et al., 2013).

Whereas the acute effects of MPH on impulsivity are well known, the long-term effects have received little attention. This is surprising, considering the large body of research elucidating persistent effects of MPH (Andersen et al., 2002; Urban and Gao, 2015) and other psychostimulant drugs (Koob and Le Moal, 1997) on reward value. These same studies have shown the effects to depend on the stage of development at which drug exposure occurs, with particular vulnerability attributed to early adolescence. While there are no absolute boundaries to the stages of adolescence, earlier in adolescence humans and non-human animals show crucial maturation of brain regions associated with impulsivity. For example, the prefrontal cortex and its dopaminergic connections are still developing and are subject to disruption in rats during that period (Reynolds et al., 2015). So far, three studies in rats have examined the long-term effects of MPH exposure during adolescence, and none have characterized its effects with adult exposure. The three adolescent rat studies showed mixed results; one found no long-term effect (Pardey et al., 2012), and the others reported long-term reductions in basal impulsivity (Adriani et al., 2007; Leo et al., 2009). Their methodologies, however, impose caveats on their interpretation.

To observe the curtailing effects of delay on reward valuation—known in behavioral research as *delay discounting*—animal studies employ operant conditioning chambers with two levers: one delivers an immediate small reward, and the other a larger reward after varying delays (Ainslie, 1975). Typical control procedures include within-session changes in delay for the large reward, counterbalancing of lever locations, training rats for center nose-poking at the beginning of trials and most importantly, training them for the association of delay with the large reward (Evenden and Ryan, 1996). None of the three adolescent rat studies employed these control procedures. Such procedural differences could lead to contrast effects and selection bias toward one of the levers (Richards et al., 1997), obscuring the findings of the experiments. As a result, it is unclear whether MPH has any long-term effect on delay discounting.

In the present study, we examined whether MPH produces a persistent effect on the devaluation of reward by delay in rats. We used a modified version of the delay discounting paradigm commonly used in the literature in order to circumvent the potential confounds listed above. Given the specificity of psychostimulant drug effects to early developmental stages, we first tested the effects of early adolescent exposure on long-term impulsivity. In addition, we used a smaller sample to determine whether similar effects could be reproduced with adult exposure. We found that moderate doses of MPH administered during early adolescence, but not during adulthood, resulted in persistently reduced choice for the delayed larger reward, indicative of greater impulsivity.

## Methods

### Experiment 1

#### Subjects

Male Long-Evans rats (Charles River, St. Constant, QC) were housed in clear, plastic cages (44.5 × 25.8 × 21.7 cm) containing beta-chip bedding. Rats were at post-natal day (PND) 21 on arrival (*n* = *50*). Cages were kept in the Animal Care Facility (ACF) under reverse 12 h light/dark conditions (lights off at 8 a.m.), at a temperature of approximately 21°C. Rats were handled daily and enrichment was provided by the addition of shredded paper to the animals' cages. Food and water was provided *ad libitum* and the rats were housed in pairs until PND 58, at which point they were food restricted (as described below) and housed individually for the remainder of the experiment. The rats were treated in accordance with the guidelines of the Canadian Council on Animal Care and as approved by the Concordia University Animal Research Ethics Committee.

#### Apparatus

Behavioural training and testing took place in operant conditioning chambers (12.5″ × 13.5″ × 13.5″; Med Associates, Georgia, VT) placed within ventilated, sound-attenuating compartments. Each chamber was equipped with a modular food pellet dispenser and a food pellet receptacle centered between two retractable levers (Coulbourn Instruments, Whitehall, PA). A continuous infrared photobeam was horizontally mounted across the entrance of the pellet receptacle to detect nose-pokes into the receptacle. Each chamber contained a house light located at the rear of the chamber and three cue lights, one above each lever and one located above the receptacle. Responses on either lever activated the food pellet dispenser, which delivered food pellets into the receptacle. Equipment was interfaced to a computer for experimental programming and data collection using MED-PC software. Rats were placed in the chambers at approximately the same time every day, during the dark phase of the light-dark cycle, and were returned to the ACF upon completion of the sessions.

#### Methylphenidate treatment

Rats at PND 25 were randomly assigned to receive one of four different doses of MPH (1, 2, 4, or 8 mg/kg) or 0.9% isotonic saline (1 ml/kg) intraperitoneally for 10 consecutive days. This period of MPH administration spanning shortly after weaning to approximately PND 35 in rats is termed early adolescence (Spear, 2000). The animals were moved from the ACF to the laboratory for the injections once daily at 11 AM.

#### Food restriction

The rats' daily food intake was restricted to about 14 g starting 23 days after the last injection and until the end of the experiment. They were fed a combination of 45-mg chocolate-flavored sucrose pellets (Bio-Serv, Frenchtown, NJ) and standard rat chow (Harlan Laboratories, IN, USA). Rats consumed chocolate pellets during the experimental task and rat chow 2 h after task completion. The exact weight of rat chow provided was adjusted daily based on body weight and the number of pellets consumed during the task, so that each rat's weight was maintained at about 80–90% of its original weight prior to food restriction.

#### Lever-press training

Lever-press training began 26 days after the last injection, at which point the rats were 58 days old. Neurobehavioural characteristics and developmental changes typical to adolescents can be seen until PND 55 in male rats, and it is recommended that PND 60 be used as a generous estimate to mark the onset of adulthood (Spear, 2000). Here, rats were trained to perform lever responses for sucrose pellets on a fixed-ratio 1 schedule for reinforcement. Each session began with the random extension of one of the two levers, and illumination of the cue light associated with the extended lever. Responses on the lever resulted in the simultaneous retraction of the lever, extinguishing of the cue light above the lever, illumination of the cue light located above the pellet receptacle, and delivery of a food pellet. Each subsequent lever extension during the session was random so that rats had approximately equal exposure to both levers. The criterion for training was set at a minimum of 60 lever responses in 1 h.

#### Nose-poke training

Once rats reached the lever-press training criterion, they were trained to nose-poke in the pellet receptacle to trigger lever presentations. This ensured that the rats were positioned centrally between the two levers at the start of each trial. Trials began with the illumination of the house light and receptacle cue light. With each successful nose poke, the receptacle cue light extinguished, one random lever cue light illuminated and its associated lever extended. Responding on the lever initiated the simultaneous retraction of the lever, extinction of the house light and lever cue light, activation of the receptacle cue light, and delivery of a food pellet. The trial ended when the rat poked his nose in the pellet receptacle, causing all lights in the box to turn off for an inter-trial interval of 15 s. After two 2 h sessions, all rats moved on to the delay discounting task.

#### Delay discounting task

The delay discounting task was modeled after Evenden and Ryan (1996). The task consisted of a discrete-trials choice procedure in which one lever was paired with the immediate delivery of one food pellet, and the second lever was paired with the delivery of four food pellets presented either immediately or after a delay. The lever corresponding to the larger outcome was consistently paired with a cue light, while the lever corresponding to the smaller outcome had no uniquely associated stimulus. Once the lever was pressed the cue light extinguished. To prevent rats from associating a particular lever with reward or delay, lever-outcome pairing changed at random across blocks of trials. Each training session consisted of 6 blocks of 14 trials each, with the first block taken as a practice round. Each block began with a pair of forced-choice trials, where the levers were extended one at a time, so that rats had no choice between outcomes. These initial trials allowed rats to learn the lever-outcome pairings for that block while breaking any stereotypy in lever choices. Next, rats underwent 12 free-choice trials, where both levers were presented simultaneously, so that rats could choose between the two outcomes. The inter-trial interval corrected for the delay of the chosen outcome, such that the beginning of one trial and the next were always 73 s apart. Rats were first trained without delay for six identical blocks. Once the animals showed almost exclusive choice of the large reward, a delay was introduced before its delivery, increasing in length with each block (0.1, 4, 10, 25, and 63 s). Lever responses in each block were checked daily until stable behavior was observed for 10 consecutive days. The minimum period of training for this phase was set at 21 days. Only the last 5 days of stable behavior were used for analysis.

### Experiment 2

Using PASS (v.15) and the large effect sizes obtained from Experiment 1, a sample size calculation indicated that for a desired power of 0.9 and including inflation for potential drop-outs, a lower sample size per group would be sufficient (*N* = 5). Thus, to test the long-term effects of MPH exposure in adults on delay discounting, 20 rats (PND = 58 on arrival) were used. Since no effect of 1 mg/kg of MPH was observed in adolescent rats, adult rats were randomly assigned to receive one of three different doses of the drug (2, 4, or 8 mg/kg) or 0.9% isotonic saline (1 ml/kg). Drug administration began at PND 60. As in Experiment 1, lever-press training began 26 days after the last injection. All other procedures were the same as in Experiment 1.

### Statistical analysis

For all analyses, lever choices within a session were quantified as the number of choices on the large reward-lever divided by the total number of lever choices. This yields the ratio *V*, known as the discounted value of the delayed reward. Behavioural stabilization during training was assessed using intraclass correlations. A rat's lever choices were considered stable when the intraclass correlation over 5 days exceeded 0.75. This value was obtained by considering the typical reliability scores in delay discounting procedures and leaving room for fluctuations in choice behavior. In Experiment 1, 10 rats failed to reach a stable performance and were removed from the study; three were removed in Experiment 2. The final analysis for Experiment 1 was based on 40 rats divided into the five injection conditions: 1 (*n* = 10), 2 (*n* = 9), 4 (*n* = 8), and 8 (*n* = 7) mg/kg of MPH, and vehicle (*n* = 6). For Experiment 2, the final analysis included 17 rats divided into 4 injection conditions: 2 (*n* = 4), 4 (*n* = 5), and 8 (*n* = 4) mg/kg of MPH, and vehicle (*n* = 4).

Using MATLAB (Mathworks, R2012b), lever choice averages for each rat across the 5 days were fit to the delay discounting equation, to estimate the rate at which the value of the delayed reward was discounted:

(1) $$V=\frac{1}{1+kd^{b}}$$

where *V* is the discounted value of the delayed reward (obtained from the lever choice averages), *d* is the delay until reward delivery, *b* is the discounting exponent reflecting the shape of the curve and *k* is the discounting rate (Rachlin, 2006).

Results from the Levene's test demonstrated a violation of the homogeneity of variance assumption for the derived *k* parameter. Thus, all significance testing was carried out on natural log-transformed *k*-values, which showed homogeneity of variance. Data were analyzed using a between-subjects ANOVA in SPSS (IBM SPSS Statistics, version 20). The cut-off point for statistically significant results was set at α = 0.05. The between-subjects variable was the *drug dose* with five factors in Experiment 1 (0, 1, 2, 4, and 8 mg/kg MPH during adolescence) and four factors in Experiment 2 (0, 2, 4, and 8 mg/kg MPH during adulthood). The magnitude of the effect was calculated using partial η^2^. Cohen's d effect sizes were calculated to compare whether each group exposed to MPH was meaningfully different from the control group.

## Results

### Experiment 1

**Figure 1:**
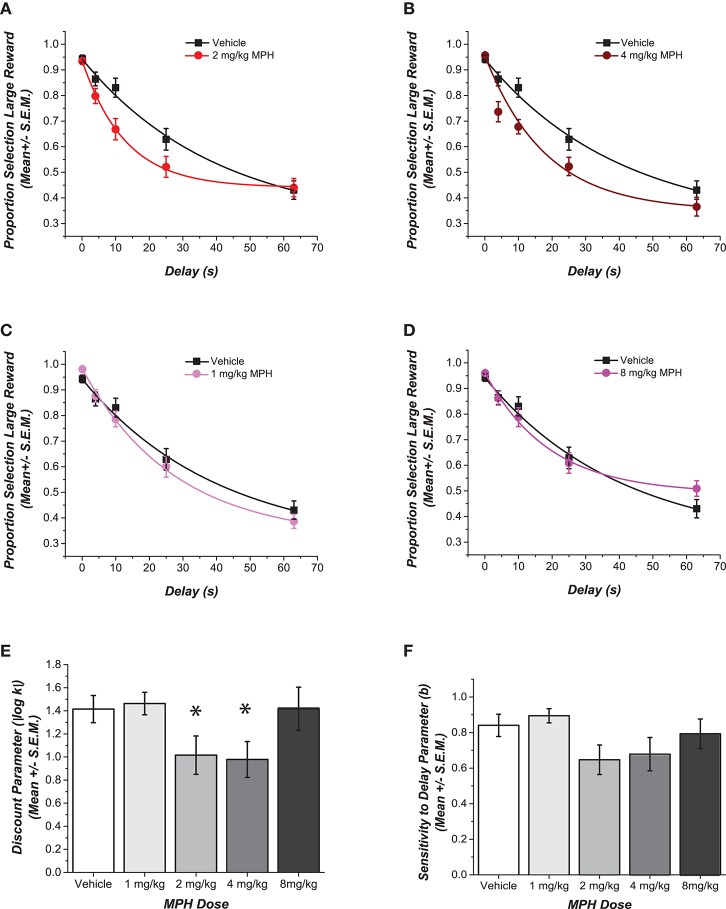
Effect of adolescent pre-treatment with MPH on delay discounting. **(A–D)** Data for each MPH-pretreated group is plotted against the vehicle to depict the dose dependent differences in choice for the delayed larger reward. Data points show the mean (± SEM) proportional choice of the large reward. The curve shows the fit of Rachlin's power function to those points. **(E,F)** Magnitudes of the shifts in the two free parameters of the function are contrasted in the bar graphs. A statistically significant main effect of dose was observed (*p* < 0.05) for the log transformed discounting rate *k*. ^*^Cohen's *d* effect sizes compared to the saline group were .98 and 1 SD for groups that received 2 and 4 mg/kg respectively. No statistically significant difference was observed in the discounting exponent *b*. Error bars represent SEM.

Choices of the large reward at increasing delays for each MPH-pretreated group are compared to the control group in Figures 1A–D. The figures also illustrate the fit of these data points to the delay discounting equation. Of the two free parameters in the delay discounting equation, only the discounting rate *k* was significantly different across groups. Specifically, different doses of MPH given intraperitoneally during adolescence had a statistically significant effect on how quickly delay decreased reward value [*F*_(4, 35)_ = 2.73, *p* = 0.045, η^2^ = 0.24; see Figure 1E]. Cohen's d effect sizes were calculated to compare the discounting rate of groups that received each dose of MPH with the group of rats that received saline during adolescence. Rats that received 1 mg/kg of MPH and 8 mg/kg of MPH showed an effect that was small in magnitude (*d* = 0.13 and *d* = 0.33, respectively). On the other hand, rats that had received moderate doses of MPH (2 and 4 mg/kg) showed a robust effect with higher discounting rates compared to the saline group (*d* = 0.98 and *d* = 1.00, respectively). Rats showed an increased discounting rate compared to their vehicle counterparts, long after exposure to the drug had discontinued.

Figure 1F shows that no difference was observed for the other free parameter, the discounting exponent *b* [*F*_(4, 35)_ = 1.88, *p* = 0.13, η^2^ = 0.08]. There were no statistically significant differences across the groups in the number of sessions until stable performance was reached [*F*_(4, 35)_ = 1.54, *p* = 0.21, η^2^ = 0.15], and there were no differences across the groups between the choice of the larger reward compared to the smaller reward when there was no delay to the larger reward [*F*_(4, 35)_ = 1.66, *p* = 0.18, η^2^ = 0.16]. Finally, there were no statistically significant differences across groups in the weight of the rats at the time of testing [*F*_(4, 35)_ = 2.81, *p* = 0.88, η^2^ = 0.03].

### Experiment 2

**Figure 2:**
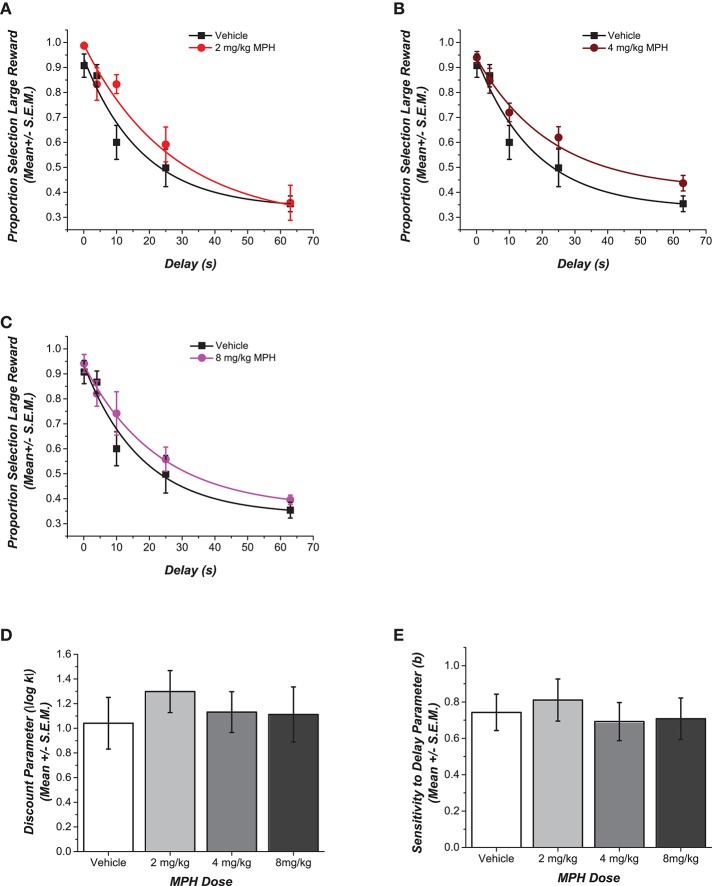
Effect of adult pre-treatment with MPH on delay discounting. **(A–C)** Data for each MPH-pretreated group is plotted against the vehicle to depict the dose dependent differences in choice for the delayed larger reward. Data points show the mean (± SEM) proportional choice of the large reward. The curve shows the fit of Rachlin's power function to those points. **(D,E)** Magnitudes of the shifts in the two free parameters of the function are contrasted in the bar graphs. No statistically significant main effect of dose was observed for the different groups in their discounting rate *k* or in the discounting exponent *b* (*p* > 0.05).

Choices of the large reward at increasing delays for each MPH-pretreated group are compared to the control group in Figures 2A–C. The figures also illustrate the fit of these data points to the delay discounting equation. Unlike those rats that were exposed to MPH during adolescence, rats that were treated with the drug during adulthood were resilient to the different doses of MPH [*F*_(3, 13)_ = 0.30, *p* = 0.83, η^2^ = 0.06). Figure 2D shows no statistically significant differences in the discounting rate *k*. Rats that received higher doses of 4 mg/kg of MPH and 8 mg/kg of MPH showed effects that were small in magnitude (*d* = 0.20 and *d* = 0.16, respectively). Rats that had received the lower dose of 2 mg/kg MPH showed a relatively greater effect with a lower discounting rate, on average, compared to the saline group (*d* = 0.74). The decrease in the discounting rate indicates more self-controlled behavior, although this was not statistically significant.

As in Experiment 1, Figure 2E shows that rats across the groups had similar discounting exponents *b* [*F*_(3, 13)_ = 0.24, *p* = 0.86, η^2^ = 0.02]. There were no statistically significant differences across the groups in the number of sessions until stable performance was reached [*F*_(3, 13)_ = 0.21, *p* = 0.21, η^2^ = 0.05], and there were no differences across the groups between the choice of the larger reward compared to the smaller reward when there was no delay to the larger reward [*F*_(3, 13)_ = 1.70, *p* = 0.22, η^2^ = 0.28]. Finally, there were no statistically significant differences across groups in the weight of the rats at the time of testing [*F*_(3, 13)_ = 1.19, *p* = 0.35, η^2^ = 0.22].

## Discussion

We have demonstrated that MPH exposure during early adolescence can persistently speed the devaluation of reward by delay. Rats that were exposed to moderate doses of MPH during adolescence showed a quicker devaluation of reward with increasing delays compared to rats that did not receive the drug. Importantly, this effect was observed long after the cessation of drug treatment.

The persistent effects of MPH observed here contrast with previously published work (Adriani et al., 2007; Leo et al., 2009; Pardey et al., 2012), a discrepancy that may be explained by methodological differences. Our methodology was modeled after one of the main paradigms used in the literature on delay discounting. In doing so, we introduced safeguards to reduce the risk of selection bias and carryover effects arising from the methodologies used previously. Specifically, the delay to the larger reward was varied within-session; the operant responses required to make a choice changed spatial locations randomly across blocks of trials; a center nose-poke ensured that the animal was equidistant from both levers before making a choice; and finally, rats were trained on the paradigm extensively until their behavior was stable.

Using this paradigm, we found that MPH accelerated the rate of reward devaluation with delay. The speed at which rats learned the task and their preference for the larger reward when it was not delayed did not differ with their drug history. Accelerated devaluation may then stem from one or more of the following: a nonlinear change in the valuation of reward such that the difference between one and four pellets was no longer the same; an increase in the perception of elapsed time; or a change in the process through which reward value and elapsed time are combined. At present, there is evidence that adolescent exposure to MPH results in long-term desensitization to natural rewards (Bolaños et al., 2003; Carlezon et al., 2003) and to drugs of abuse (Andersen et al., 2002; Vendruscolo et al., 2008; Crowley et al., 2014), suggesting that a change in reward valuation is responsible. However, the enduring effects of adolescent exposure to MPH on time perception, timed performance and its combination with reward value also deserve to be evaluated in future studies.

This study also provides experimental evidence of a nonlinear effect of MPH dose on impulsivity. Whereas doses of 2 and 4 mg/kg of MPH increased impulsivity, a lower (1 mg/kg) and a higher (8 mg/kg) dose showed no effect on the behavior. Non-linear effects at these doses are also visible in the prefrontal cortex, an area known to play an important role in delay discounting (Winstanley, 2010). Doses of MPH shown effective in this study also increase firing rates in the prefrontal cortex (Devilbiss and Berridge, 2008), facilitate long-term potentiation *in vivo* (Burgos et al., 2015) and increase glutamate signaling and surface expression of several subtypes of N-methyl-D-aspartate receptors (Cheng et al., 2014). In contrast, higher doses of MPH (greater than 5 mg/kg) have the opposite effects in all four cases (Devilbiss and Berridge, 2008; Cheng et al., 2014; Burgos et al., 2015). The long-term behavioral effects of MPH we describe may then have a neural basis in the prefrontal cortex.

The impulsivity-promoting effect of MPH is specific to exposure during adolescence, a period of continued neural development in the prefrontal cortex (Giedd et al., 1999; Gogtay et al., 2004; Winstanley, 2010) as well as in the connected mesocortical dopamine system (Benes et al., 1996; Manitt et al., 2011; Naneix et al., 2012; Reynolds et al., 2015). Conspicuously, dopamine connectivity has been linked to the computation of temporal influence in the subjective valuation of reward (Pine et al., 2010; Winstanley, 2010), and MPH exposure during adolescence produces an array of persistent effects on the midbrain dopamine system. Among them, dopamine neural activity in the VTA is decreased (Brandon et al., 2003), as is the density of the dopamine transporter in the striatum. This latter effect may be due to a change in connectivity and morphology of the dopamine axons in the striatum and prefrontal cortex (Moll et al., 2001). Such downregulation in the dopaminergic system is associated with impulsive choice (Kheramin et al., 2004; Zeeb et al., 2010; Hernandez et al., 2014). Notably, the aforementioned changes in connectivity and neurotransmission in the prefrontal cortex and mesocortical dopamine system are not observed when MPH exposure occurs during adulthood (Moll et al., 2001; Brandon et al., 2003; Somkuwar et al., 2013; Crowley et al., 2014; van der Marel et al., 2014). Likewise, our findings show that although adolescent exposure to moderate doses of MPH increased impulsivity, the same did not hold true when exposure occurred during adulthood. In our adult-exposure sample, no effect of the drug was observed and if anything, a trend in the opposite direction was detected where rats that had been exposed to certain doses (2 and 4 mg/kg) seemed to exert more self-control. In light of these correlations, it is possible that the stage-specific effect of MPH on delay discounting is mediated by alterations in the development of the mesocortical dopaminergic pathway.

In sum, adolescence may constitute a critical period in the development of the system underlying delay discounting—a period in which the system is acutely susceptible to environmental influences such as psychostimulant exposure, enabling lasting changes in impulsive behavior.

## Author contributions

GH and AA conceived the study. ZA, GH, and AA designed the study. ZA and AS obtained the data. ZA, AS, and GH analyzed the data. ZA, GH, and AA wrote the paper.

### Conflict of interest statement

The authors declare that the research was conducted in the absence of any commercial or financial relationships that could be construed as a potential conflict of interest.
